# Quantitative SPECT/CT parameters of myocardial ^99m^Technetium-3,3-diphosphono-1,2-propanodicarboxylic acid (DPD) uptake in suspected cardiac transthyretin amyloidosis

**DOI:** 10.1186/s13550-021-00828-0

**Published:** 2021-09-06

**Authors:** Simona Ben-Haim, A. Chicheportiche, E. Goshen, M. Arad, M. Smekhov, L. J. Menezes, P. M. Elliott, E. O’Mahoney, E. Stern, Bella Yuzefovich, J. B. Bomanji

**Affiliations:** 1 Hadassah Medical Center, Hebrew University, Jerusalem, Israel; 2 NIHR Biomedical Research Centre, UCL Institute of Nuclear Medicine, London, UK; 3 Wolfson Medical Center, Sackler School of Medicine, Tel Aviv University, Tel Aviv, Israel; 4 Chaim Sheba Medical Center, Ramat Gan, Israel; 5 GE Healthcare, Haifa, Israel

**Keywords:** Amyloid heart disease, SPECT, Image analysis, SPECT/CT quantitation

## Abstract

**Background:**

^99m^Tc-labelled bisphosphonates are used for imaging assessment of patients with transthyretin cardiac amyloidosis (ATTR). Present study evaluates whether quantitative SPECT/CT measurement of absolute myocardial ^99m^Tc-labelled 3,3-diphosphono-1,2-propanodicarboxylic acid (Tc-DPD) uptake can diagnose patients with suspected ATTR.

**Methods:**

Twenty-eight patients (25 male, age 80.03 ± 6.99 years) with suspected ATTR referred for Tc-DPD imaging had planar and SPECT/CT imaging of the chest. Three operators independently obtained Tc-DPD myocardial SUVmax and SUVmean above threshold (SMaT) (20, 40 and 60% of SUVmax), using a semi-automated threshold segmentation method. Results were compared to visual grading (0–3) of cardiac uptake.

**Results:**

Twenty-two patients (78%) had cardiac uptake (2 grade 1, 15 grade 2, 5 grade 3). SUVmax and SMaT segmentation thresholds enabled separating grades 2/3 from 0/1 with excellent inter- and intra-reader correlation. Cut-off values 6.0, 2.5, 3 and 4 for SUVmax, SMaT_20,40,60,_ respectively, separated between grades 2/3 and 0 /1 with PPV and NPV of 100%. SMaT_20,40,60_(cardiac)/SUVmean (liver) and SMaT_20,40,60_(cardiac)/SUV_mean_(liver/lung) separated grades 2 and 3.

**Conclusion:**

Quantitative SPECT/CT parameters of cardiac Tc-DPD uptake are robust, enabling separation of patients with grades 2 and 3 cardiac uptake from grades 0 and 1. Larger patient cohorts will determine the incremental value of SPECT/CT quantification for ATTR management.

## Introduction

Cardiac amyloidosis is characterized by protein misfolding and myocardial deposition mainly of monoclonal light chain (AL) or transthyretin (ATTR), resulting in restrictive cardiomyopathy and heart failure [1, 2].

ATTR amyloidosis may be acquired, associated with wild-type transthyretin (TTR), or hereditary, associated with TTR gene variants. Cardiac ATTR amyloid deposits are present in up to 25% elder individuals [3], more common among patients with heart failure and preserved ejection fraction (HFpEF) [4].

^99m^Technetium (Tc)-labeled bisphosphonates used for bone scintigraphy including ^99m^Tc-3′3-diphosphono-1,2-propanodicarboxylic acid (DPD), pyrophosphate (PYP) and hydroxy-dimethylene diphosphonate (HDP) localize to cardiac amyloid deposits [2, 5–8] and can identify cardiac ATTR amyloid deposits early in the course of disease, sometimes prior to echocardiography or magnetic resonance (MRI) [9] and may obviate the need for endomyocardial biopsy [10].

Planar and SPECT images interpreted visually, by grading the intensity of myocardial uptake as compared to skeletal activity or by semi-quantitative techniques, measuring heart/whole body retention or heart/contralateral lung uptake on planar scans and have high sensitivity and specificity in cases with either intense or absent uptake, but are less accurate in equivocal radiotracer activity [2, 5–7, 10, 11].

New emerging therapies either prevent TTR amyloid formation [12, 13] or inhibit TTR expression [14], with promising results. A sensitive and accurate method to quantify amyloid burden is needed to evaluate treatment response. Quantification of myocardial Tc-DPD uptake may overcome shortcomings of planar studies in borderline cases and may improve the diagnostic performance, monitor the amyloid burden over time and determine ATTR treatment response.

Initial reports of SPECT quantitation show encouraging preliminary results [15–21]. Present study assesses the potential of SPECT/CT quantification of Tc-DPD myocardial uptake in patients with suspected cardiac amyloidosis.

## Methods

### Patients

This retrospective two center international study includes 30 consecutive patients with cardiomyopathy and suspected cardiac amyloidosis based on symptoms, monoclonal protein studies, echocardiography and/or MRI. Biopsies were performed in 14 patients, including 6 endomyocardial, and genetic testing in 2 cases. Patients were referred for Tc-DPD SPECT/CT for routine clinical work-up between May 2013 and June 2017 at Chaim Sheba Medical Center, Israel and University College London Hospitals, UK. The study received IRB approval in both institutions and the need for informed consent was waived.

### ^***99m***^***Tc-DPD studies***

Planar whole body imaging was acquired at 5 and 180 min after i.v. injection of 743 ± 102 MBq of Tc-DPD using a SPECT/CT (Discovery 670, GE Healthcare) with low energy, high resolution collimators for a scan speed of 13 cm/min. SPECT/CT of the chest was performed with 180° L-mode SPECT, from 45° LAO to 45° LPO. Step-and-shoot mode data were acquired every 3°, 30 s/step, with two 15-s frames in each step. CT parameters were 120 kV, 120 mA, slice thickness 2.5 mm. Data reconstruction used OSEM (4 iterations, 10 subsets) and were corrected for motion, attenuation, scatter and collimator blurring. Attenuation correction quality control ascertained good registration of SPECT and CT.

*Visual assessment*: Cardiac Tc-DPD uptake was assessed visually on planar scintigraphy and SPECT by Nuclear Medicine physicians according to the expert consensus recommendations [22], scored as grade 0—absent cardiac, normal skeletal uptake; grade 1—mild cardiac uptake, inferior to rib; grade 2—moderate cardiac uptake equal to rib; grade 3—high cardiac uptake, mild or absent rib uptake.

**Fig. 1:**
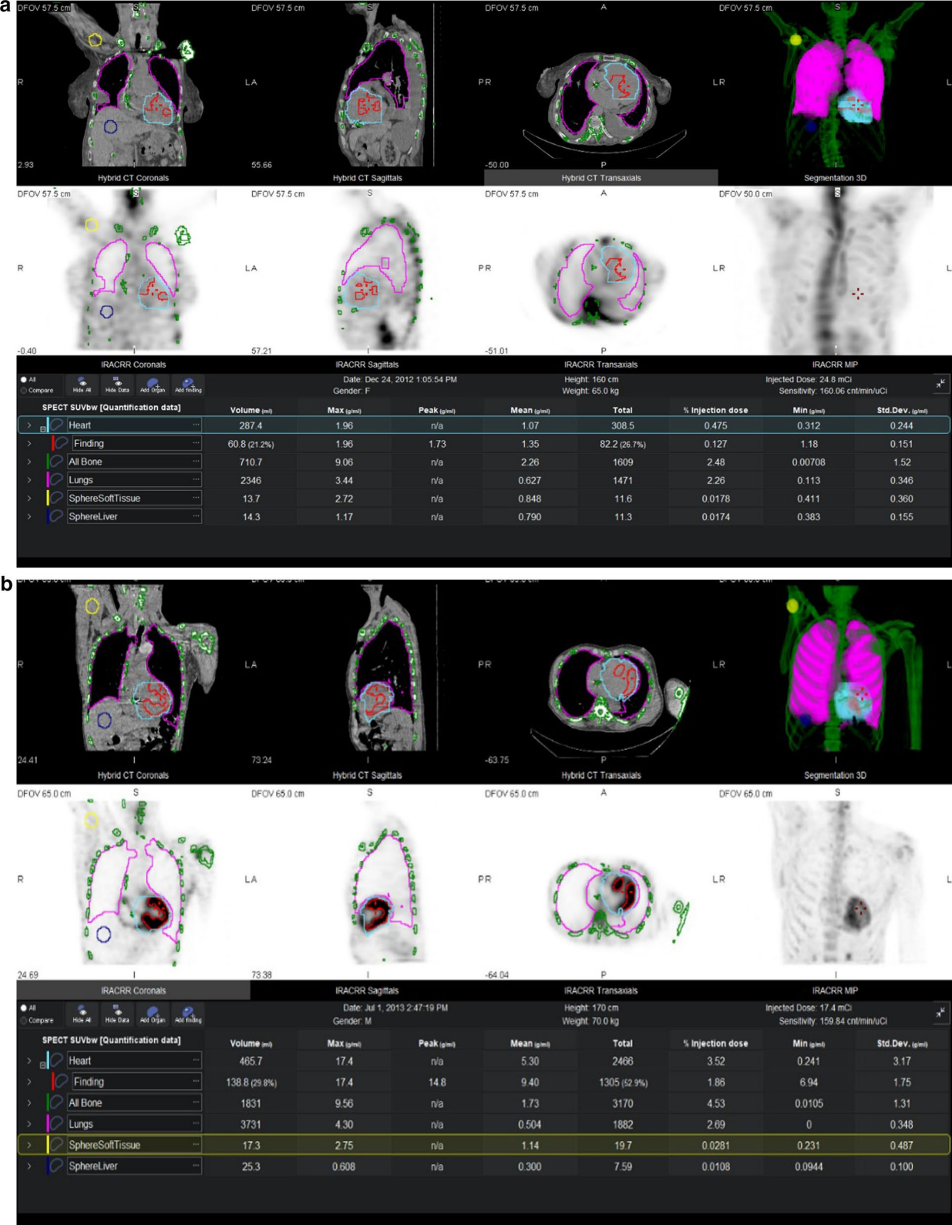
SPECT/CT segmentation in patients with **a** no cardiac activity (grade 0) and **b** abnormal cardiac activity greater than adjacent ribs (grade 3). LV myocardium (cyan—Master VOI based on CT image; red VOI—automatic generated, containing all voxels within master VOI having ≥ 20% of max SUV), bone (green VOI), lungs (pink VOI), liver (purple sphere VOI) and soft tissue (yellow sphere VOI) using Q Volumetrix MI. Display of quantitative analysis results in table (bottom of figure). LV, left ventricle; VOI, volume of interest

*Quantitative assessment*: Quantification of Tc-DPD uptake was performed using Q.Volumetrix MI on a Xeleris workstation version 4 DR (GE Healthcare), given information of radioisotope, dose and time of injection. Tracer uptake (MBq/ml) or percent of injected dose (%ID) are calculated in a volume of interest (VOI), generated automatically or interactively by the user. Standardized uptake values (SUV) are calculated adding patient height and weight (Fig. 1).

*Cardiac uptake*: Segmentation of Tc-DPD uptake in the left ventricle (LV) wall was performed by drawing boundaries (a “Master VOI”) based on the CT images of the co-registered SPECT-CT study (cyan lines, Fig. 1). The Master VOI was automatically projected to the co-registered SPECT images. The Master VOI of the left ventricle based on non-enhanced CT images includes also the lumen. This may lead to inclusion of blood pool uptake when projecting the master VOI on the SPECT images. Therefore, a threshold-based method was applied for segmenting Tc-DPD uptake in the corresponding SPECT images, as follows: within the Master VOI, SUVmax was automatically detected by the Q.volumetrix MI software. Then, 3 threshold value were applied to segment uptake at 20%, 40% and 60% of SUVmax. All voxels containing SUV values above the threshold inside the Master VOI were automatically segmented (red lines, Fig. 1). In addition to SUVmax, an average (mean) of SUV values in voxels selected by the threshold-based segmentation was calculated automatically by the Q Volumetrix MI software (SUV Mean above threshold—SMaT_20_, SMaT_40_ and SMaT_60_ for 20%, 40% and 60% of SUVmax, respectively)_._ Values obtained by applying SMaT_40_ are depicted in line 2, table of Fig. 1). The exploration of 3 threshold values was driven by the need to find the most appropriate threshold approach (e.g. that would most accurately separate between grades of myocardial uptake).

Inter-observer variability was assessed by three operators who performed quantitative analysis for each study. One operator repeated analysis of all studies after four weeks, to assess intra-reader reproducibility.

*Uptake in bone, lung, liver and soft tissue*: Bone and lung SUVmean were calculated on SPECT inside segmented volumes obtained from an automatic CT-HU threshold-based segmentation algorithm (green and magenta lines respectively, Fig. 1).

For liver and soft tissue SUVmean 25 ml and 15 ml spheres were placed by operator in liver (blue lines, Fig. 1) and shoulder muscle (yellow lines, Fig. 1), respectively.

Cardiac SUV_max_, SMaT_20_, SMaT_40_ and SMaT_60_ were normalized to SUVmean of lungs, liver, bones and soft tissue, to SUVmean liver/lungs and to SUVmean bone/soft tissue.

### Statistical analysis

Continuous variables are presented as mean ± standard deviation (SD). Differences between datasets separated by grade of myocardial uptake were assessed with Mann–Whitney *U* test. *p* < 0.05 was statistically significant.

SUVmax and SMaT that could separate between grades of myocardial uptake were defined as cut-off value with highest true positive rate (TPR) and lowest false positive rate (FPR). Positive and negative predictive value (PPV, NPV) were also calculated.

Inter-reader correlation was calculated using Intraclass Correlation Coefficient (ICC, MedCalc Statistical Software version 18.11.3, Ostend, Belgium). Intra-reader reproducibility was calculated by the Bland–Altman method.

## Results

The study included 30 patients. One patient with grade 2 uptake and one patient with grade 3 uptake and chronic renal failure who required dialysis and had incomplete data were excluded from the quantitative analysis. Of the remaining 28 patients (25 male, age 80.03 ± 6.99 years) 22 patients (78%, 20 male, age 80.04 ± 7.26 years) had Tc-DPD cardiac uptake. Two had grade 1, 15 grade 2 and five had grade 3 uptake. Six patients had no cardiac uptake (grade 0). ATTR was diagnosed in 21/22 patients with positive Tc-DPD studies and was excluded in one patient with known AL amyloidosis (Table 1).

**Table 1:** Summary of findings in patients with ^99m^Tc-DPD cardiac uptake

Pt	M/F	Age	Clinical	Serum studies	DPD		Biopsy	Gen test	Diagnosis
					Grade	SUVmax			
1	M	75	Pos	Neg	2	17.4	Pos fat	NA	ATTR
2	M	80	Pos	Neg	2	14.0	NA	NA	ATTR
3	M	60	Pos	Neg	2	12.3	NA	NA	ATTR
4	M	74	Pos	Neg	3	18.1	Pos fat	NA	ATTR
5	M	80	Pos	Neg	2	14.3	NA	NA	ATTR
6	M	80	Pos	Mon gamm	2	13.6	Pos EM	NA	AL
7	F	71	Pos	Neg	1	2.2	Pos BM	Mut	ATTR
8	M	81	Pos	Neg	2	13.1	NA	NA	ATTR
9	M	68	Pos	Neg	1	3.3	Pos Bowel	Mut	ATTR
10	M	84	Pos	Neg	3	13.2	NA	NA	ATTR
11	M	85	Pos	Neg	2	11.0	NA	NA	ATTR
12	M	74	Pos	Neg	2	17.6	NA	Neg	ATTR
13	F	89	Pos	Mon LC	2	22.3	Neg BM, fat	NA	ATTR senile
14	M	82	Pos	Neg	2	15.2	Pos BM	NA	ATTR
15	M	88	Pos	Neg	3	10.4	NA	NA	ATTR
16	M	85	Pos	Neg	2	18.0	NA	NA	ATTR
17	M	89	Pos	Neg	2	9.2	NA	NA	ATTR
18	M	82	Pos	Neg	2	18.1	NA	NA	ATTR
19	M	83	Severe AS	Neg	2	13.6	NA	NA	ATTR
20	M	85	Pos	Neg	2	16.6	Pos muscle	NA	ATTR
21	M	79	Pos	Neg	2	8.6	NA	NA	ATTR
22	M	87	Pos	Neg	2	10.4	NA	NA	ATTR

M, male; F, female; Grade, visual; SUVmax, SUV (body weight) max (g/ml); Gen test, genetic testing; pos, positive; neg, negative; ATTR, cardiac transthyretin amyloidosis; AL, light chain amyloidosis; EM, endomyocardial; BM, bone marrow; MUT, mutation Se77Tyr; Mon Gam, monoclonal gammopathy; Mon LC, monoclonal light chains; AS, aortic stenosis

SPECT/CT measured cardiac SUVmax in patients with grade 0, 1, 2 and 3 was 1.85 ± 0.26, 2.79 ± 0.53, 14.05 ± 3.07, 15.26 ± 4.34, respectively (Table 2). There was a statistically significant difference between grades 0 and 1 versus grade 2, (*p* < 0.001 and *p* = 0.01, respectively) and between grades 0 and 3 (*p* = 0.004) (Table 3). In grade 0 cardiac SMaT_20_, SMaT_40_ and SMaT_60_ was 0.78 ± 0.25, 0.98 ± 0.2 and 1.31 ± 0.22 g/ml, respectively. These values were lower than the corresponding values in patients with grade 1, although not statistically significant (Table 3). Cardiac uptake SMaT_20_, SMaT_40_ and SMaT_60_ for grade 2 and 3 were not significantly different but were significantly higher compared to the same values in grades 0 and 1 (*p* = 0.01) (Table 3).

**Table 2:** Summary of visual and quantitative findings

Grade	No pts	SUV_max_				SUVmean_20_*				SUVmean_40_*				SUVmean_60_*			
		Mean	SD	Min	Max	Mean	SD	Min	Max	Mean	SD	Min	Max	Mean	SD	Min	Max
0	6	1.85	0.26	1.33	2.27	0.78	0.25	1.48	1.12	0.98	0.20	0.72	1.22	1.31	0.22	0.99	1.53
1	2	2.79	0.53	2.25	3.32	1.25	0.20	1.11	1.39	1.49	0.31	1.27	1.71	1.96	0.53	1.59	2.34
2	15	14.05	3.07	8.60	18.10	5.98	1.45	3.69	8.44	8.14	1.98	4.86	11.3	10.23	2.65	6.05	15.4
3	5	15.26	4.34	10.40	22.30	5.94	1.38	4.47	8.11	7.62	1.65	5.88	10.33	9.47	1.92	7.41	12.53
Total	28																

Pts, patients; SUV, standardized uptake value; SUVmean_20_, SUVmean using a segmentation threshold of 20% SUVmax; SUVmean_40_, segmentation threshold of 40%SUVmax; SUVmean_60_, segmentation threshold of 60% SUVmax

*Values are mean uptake above threshold; SD, standard deviation; min, minimum; max, maximum

**Table 3:**
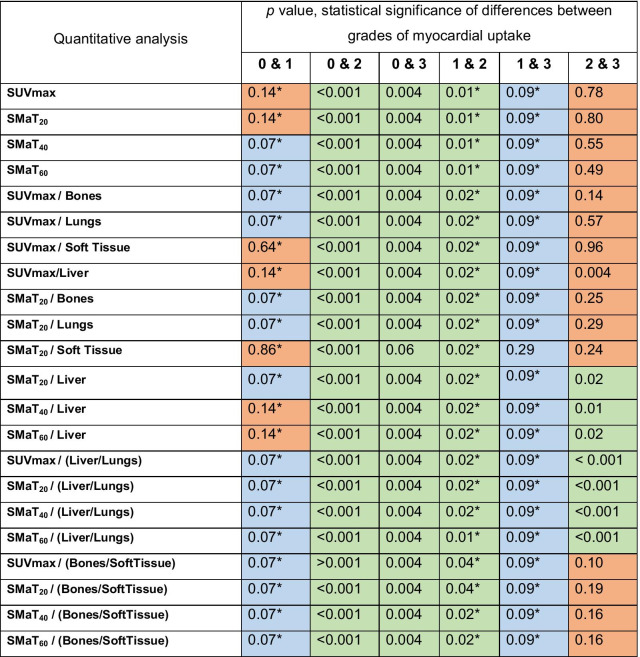
Statistical significance of the difference in SUV values between grades of myocardial uptake

SUV, standardized uptake value; SMaT_20_, SUV_mean_ using a segmentation threshold of 20% SUVmax; SMaT_40_, SUV_mean_ using segmentation threshold of 40%SUVmax; SMaT_60_, segmentation threshold of 60% SUVmax; Bones, SUV_mean_ bones; Lungs, SUV_mean_ lungs; Soft tissue, SUV_mean_ soft tissue; Liver, SUV_mean_ liver

Green, blue and orange cells represent p values lower than 0.05, between 0.05 and 0.1 and higher than 0.1, respectively

*Only 2 points for grade 1

Three readers’ inter-reader correlation coefficients were all above 0.99 and intra-reader correlation coefficients ranged between 0.91 and 1.0 (Table 4).

**Table 4:** Inter- and intra-operator agreement

Operators	Correlation coefficient SUVmean_20_	Correlation coefficient SUVmean_40_	Correlation coefficient SUVmean_60_
I and II	0.994926	0.998669	0.998777
II and III	0.996651	0.992594	0.998067
I and III	0.994297	0.990702	0.997411
I.1 and I.2	1	0.958495	0.910854

SUV, standardized uptake value; SUVmean_20_, SUVmean using a segmentation threshold of 20% SUVmax; SUVmean_40_, segmentation threshold of 40%SUVmax; SUVmean_60_, segmentation threshold of 60% SUVmax

^*^3 operators, I, II and III; operator I performed the analysis twice (I.1 and I.2)

**Fig. 2:**
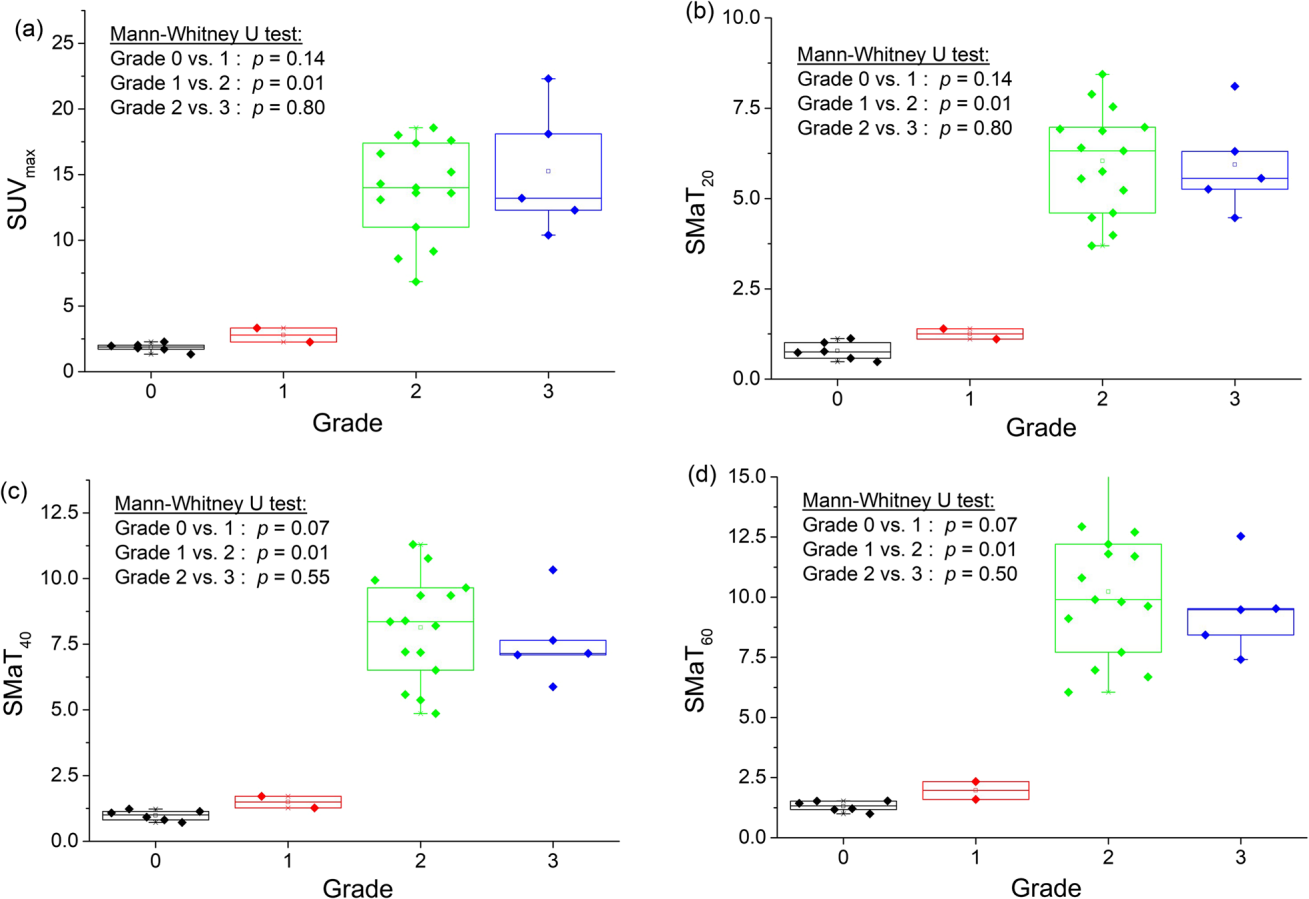
Bar graph showing separation of quantitative myocardial activity **a** SUVmax, **b** SMaT_20_, **c** SMaT_40_, **d** SMaT_60_ by visual cardiac uptake grade. SUV, standardized uptake value; SMaT_20_, SUV mean above 20% threshold; SMaT_40_, SUV mean above 40% threshold; SMaT_60_, SUV mean 60% threshold

**Fig. 3:**
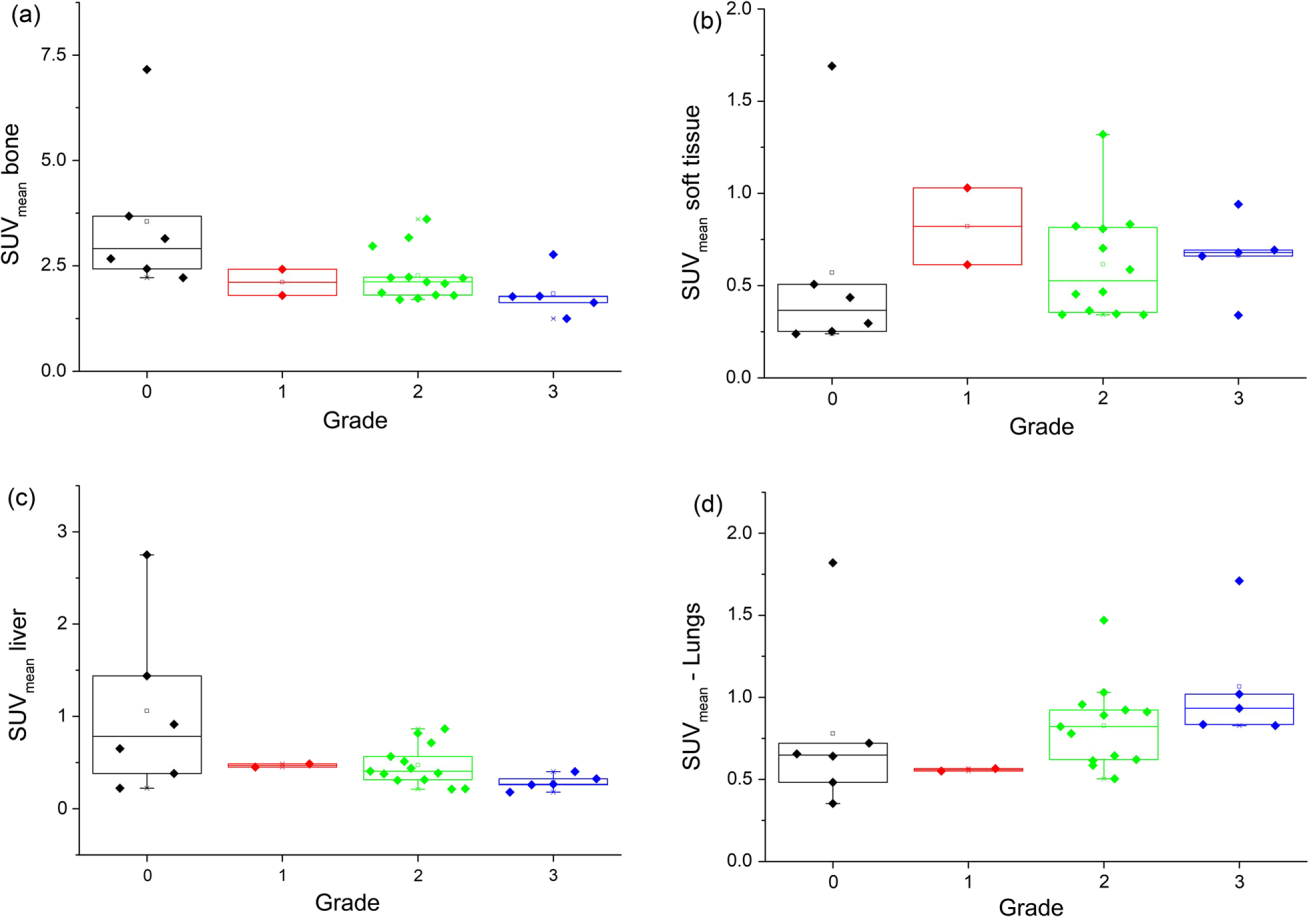
Bar graph showing separation of SUVmean of **a** bone, **b** soft tissue, **c** liver, **d** lungs by visual cardiac uptake grade. SUV, standardized uptake value

With increasing grade of myocardial uptake the activity in bone and liver decreased significantly, (*p* = 0.01, *p* = 0.03, respectively). There was a tendency for higher soft tissue and lung activity with increasing grade of myocardial uptake, but not statistically significant (*p* = 0.60 and 0.98, respectively) (Figs. 2, 3).

**Fig. 4:**
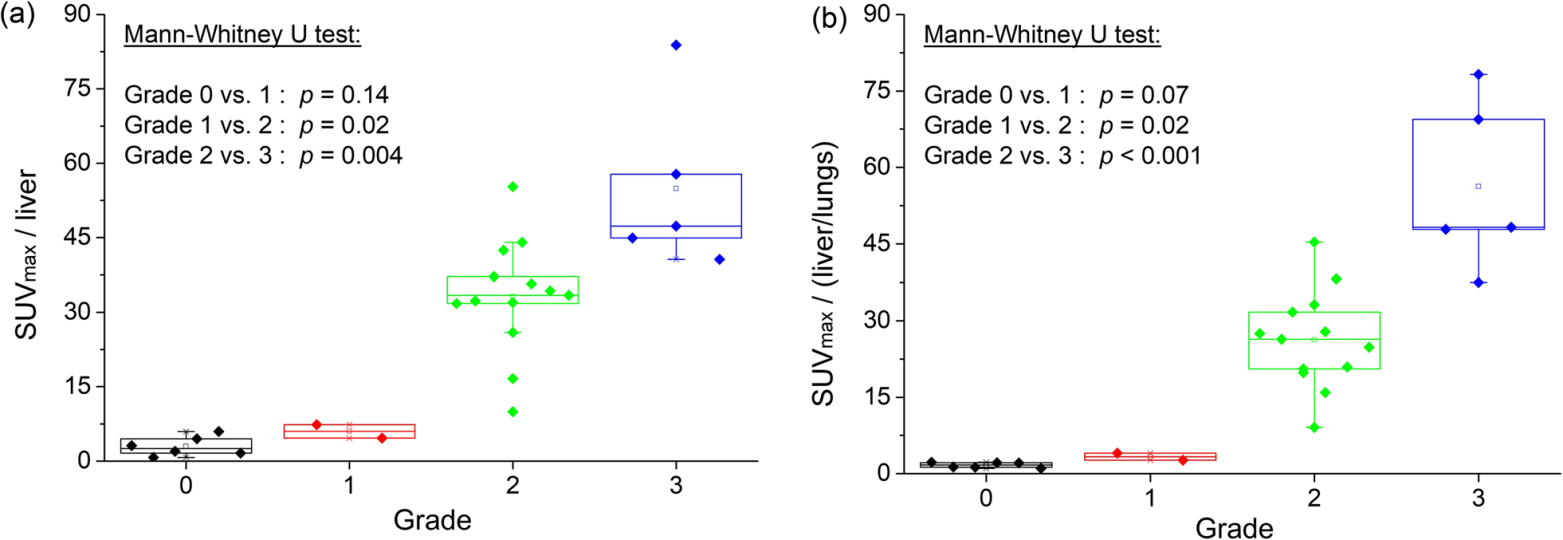
Bar graph showing separation of **a** SUVmax normalized to mean liver activity, **b** SUVmax/SUVmean liver*SUVmean lungs by visual cardiac uptake grade. SUV, standardized uptake value

Normalization of cardiac SUVmax and SMaT to SUVmean of lungs, bones and soft tissue separated studies with grades 0 and 1 from grades 2 and 3 without additional statistical significance (Table 3). SUVmax/ SUVmean liver and SMaT cardiac/SUVmean liver was significantly different for grades 2 and 3 for all (*p* = 0.004 for SUVmax, *p* = 0.02 for SMaT_20_ and SMaT_60_, *p* = 0.01 for SMaT_40_). SUVmax /(liver/lungs) and SMaT cardiac/(liver/lungs) separated between grades 2 and 3 (*p* < 0.001), between grades 0 and 2 (*p* < 0.001, all thresholds) and between grades 1 and 2 (Table 3). SMaT_40_ and SMaT_60_ improved separation between grades 0 and 1, although not statistically significant (*p* = 0.07), with similar results after normalizing to other organs (Table 3, Fig. 4).

SUVmax cut-off value of 6.0 separated between cardiac uptake grades 2 and 3 compared to 0 and 1 with FPR 0% and TPR, PPV and NPV 100%. Same results were obtained for SMaT_20_, SMaT_40_ and SMaT_60_ cut-off values of 2.5, 3.3 and 4.2, respectively.

Cardiac/(liver/lungs) defined thresholds of 41.9, 15.8, 20.2 and 24.4 for SUVmax, SMaT_20_, SMaT_40_ and SMaT_60_, respectively for separating between grades 2 and 3 with FPR of 7.7% for SUVmax, 15.4% for SMaT_20_ and SMaT_40_ and 13.3% for SMaT_60_, PPV of 80% for SUVmax and 71% for SMaT, and TPR and NPV 100% for all SMaT. For SUVmax TPR was 80% and NPV was 92.3%. SUVmax cardiac/(bone/soft tissue) and SMaT cardiac/(bone/soft tissue) for all three thresholds were significantly different between grades 0 or 1 and 2 or 3 (*p* < 0.001), but not between grades 2 and 3 (Table 3).

SUVmax cut-off of 3.0 and SMaT cut-off values of 1.3, 1.5 and 2.1 (20%, 40% and 60% thresholds, respectively) separated between grade 0 and 1/2/3 with FPR 0% and TPR, PPV and NPV of 100%.

Endomyocardial biopsy was negative in five of six patients, all with grade 0 and negative Tc-DPD. One patient with known AL amyloidosis had grade 2 cardiacTc-DPD uptake with SUVmax 13.6 and SMaT of 3.66, 4.88 and 6.05 (SMaT_20_, SMaT_40_ and SMaT_60,_ respectively). Biopsy excluded ATTR (patient 6, Table 1). Two patients with grade 1 cardiac uptake diagnosed with ATTR, mutation Ser77Tyr (patients 7 and 9, Table 1) had SUVmax values of 2.2 and 3.3 and SMaT of 1.1, 1.27, 1.59 and 1.39, 1.71 and 2.22 (SMaT_20_, SMaT_40_ and SMaT_60_,), respectively.

## Discussion

Endomyocardial biopsy is the gold standard for diagnosing ATTR, but does not provide information regarding disease extent, prognosis, progression, and response to therapy [1, 12–14].

Bone-seeking radiotracers are sensitive and specific for ATTR using a visual grading score or semi-quantitative assessment of myocardial uptake in cases with either intense or absent cardiac activity, but less accurate in equivocal cases [5–8, 10, 11, 22]. Grade 2 or 3 Tc-DPD/PYP/HMDP uptake with no monoclonal antibodies in blood and urine has specificity and PPV above 98% for diagnosis of ATTR [10].

Novel therapeutic agents recently introduced may require precise assessment of disease burden at diagnosis and during follow up [1, 17–19].

Present results in 28 consecutive patients with clinically suspected ATTR indicate that quantitative SPECT/CT measurements of Tc-DPD cardiac uptake are feasible and robust. We observed an increase in SUVmax and SMaT of cardiac Tc-DPD uptake with higher visual grade (Table 2, Fig. 2). Normalization of the cardiac SUVmax or SMaT to that of the liver and to the liver/lungs ratio separated between grades 2 and 3 Tc-DPD uptake (Table 3). The SUVmax or SMaT of cardiac/(bone/soft tissue) distinguished between grades 0/1 and 2/3 but not between grades 2 and 3. Higher cardiac SUVs correlating with increasing visual grade were previously described [15–21]. Large variations in SUVs were measured in current study for patients with both grade 2 and 3, whereas Scully et al. described similar findings only in patients with grade 2 cardiac uptake [18].

Results of quantitative assessment of extracardiac activity in present study differ from previous studies. The decrease in liver uptake with increasing cardiac activity reported here has not been previously demonstrated. The decrease in bone uptake is in agreement with some authors [18], but not all [20]. We have demonstrated that normalization of cardiac uptake to liver activity enables separation between grades 2 and 3, further enhanced by the use of the cardiac/(liver/lung) ratio. Cardiac/(bone/soft tissue) ratio previously reported to distinguish between grades 2 and 3 [18] was not confirmed in our study group. We were the first to define quantitative cut-off values to separate between grades 2/3 compared to grades 0/1 and between grade 0 and grades 1/2/3 myocardial uptake with a FPR of 0% and TPR, PPV and NPV of 100%.

High reproducibility is crucial for robust and accurate quantification, enabling precise assessment and follow up in a clinical setting. All quantitative parameters of cardiac activity were highly reproducible with excellent inter- and intra-observer agreement. High inter- [18] and intra-observer [18, 21] correlations were reported in certain previous patient subgroups.

Feasibility of quantitative SPECT/CT with Tc-DPD/HDP/PYP in patients with suspected ATTR was recently assessed using CZT-based SPECT/CT scanners [19–21] with overall similar results to conventional SPECT/CT. Bone activity showed no significant differences between the scores, whereas lung and soft tissue activity was significantly higher in grade 2 and 3 [20]. Cardiac amyloid activity (CAA), defined as cardiac SUVmean*LV volume was proposed [21]. While quantitation results are similar, CZT-based SPECT/CT may be justified by reduced radiation exposure, improved throughput and patient comfort. With increasing availability in future, CZT-based devices may play an important role for repeat studies to monitor response to therapy. This also justifies the need for standardization of quantitative analysis.

Present study has several limitations. Although a dual-center study the number of patients included in the analysis is limited, with only two grade 1 cases. Also, due to the retrospective design and reflecting clinical practice, patients did not have routinely endomyocardial biopsies. As mentioned, the Master VOI of the left ventricle based on non-enhanced CT images includes also the left ventricular lumen. This may lead to inclusion of blood pool uptake when projecting the master VOI on the SPECT images. SMaT enables to exclude voxels containing low SUV values, and may therefore overcome this limitation.

*New knowledge gained*: SPECT/CT quantitation of ^99m^Tc-DPD cardiac uptake is robust and separates grades 2 and 3 from grades 0 and 1 ATTR. Quantitative normalization to liver and lung, as well as specific cut-off values enables separation between all grades of cardiac uptake.

In patients with advanced ATTR visual and semi-quantitative heart-to-contralateral lung ratio on repeat Tc-PYP planar scans showed no significant change, despite clinical progression of ATTR [23]. Tc-DPD SPECT/CT quantification with reproducible measurements of amyloid burden may be of value for monitoring response to the newly available therapies and should be evaluated in future in large patient cohorts in order to establish normal values to be further used to define prognosis and to tailor and monitor response to treatment.

## Conclusion

Quantitative SPECT/CT parameters of cardiac Tc-DPD uptake are robust, separating grades 2 and 3 myocardial uptake from grades 0 and 1. Normalization of cardiac SUVmax or SMaT to liver SUVmean and liver/lung SUVmean ratio and specific cut-off values further separate between all grades. Evaluation in larger patient cohorts is needed to determine the added value of SPECT/CT quantification over visual assessment in diagnosis of borderline cases, in follow-up of patients with suspected ATTR, and in monitoring response to new available therapies.

## Data Availability

The datasets used and/or analysed during the current study are available from the corresponding author on reasonable request.

## Abbreviations

AL: Light chain amyloidosis
ATTR: Transthyretin amyloidosis
HFpEF: Heart failure with preserved ejection fraction
Tc-DPD: ^99M^Tc-3′3-diphosphono-1,2-propanodicarboxylic acid
PYP: Pyrophosphate
HDP: Hydroxy-dimethylene diphosphonate
VOI: Volume of interest
LV: Left ventricle
SUV: Standardized uptake value
SMaT: SUV mean above threshold
FPR: False positive rate
TPR: True positive rate
PPV: Positive predictive value
NPV: Negative predictive value

## References

[1] Ruberg FL, Berk JL (2012). Transthyretin (TTR) cardiac amyloidosis. Circulation.

[2] Quarta CC, Guidalotti PL, Longhi S (2012). Defining the diagnosis in echocardiographically suspected senile systemic amyloidosis. JACC Cardiovasc Imaging.

[3] Tanskanen M, Peuralinna T, Polvikoski T (2008). Senile systemic amyloidosis affects 25% of the very aged and associates with genetic variation in alpha2-macroglobulin and tau: a population-based autopsy study. Ann Med.

[4] Mirzoyev SA, Edwards WD, Mohammed SF (2010). Cardiac amyloid deposition is common in elderly patients with heart failure and preserved ejection fraction. Circulation.

[5] Perugini E, Guidalotti PL, Salvi F (2005). Noninvasive etiologic diagnosis of cardiac amyloidosis using 99mtc-3,3-diphosphono-1,2-propanodicarboxylic acid scintigraphy. J Am Coll Cardiol.

[6] Rapezzi C, Guidalotti P, Salvi F, Riva L, Perugini E (2008). Usefulness of Tc-99m-DPD scintigraphy in cardiac amyloidosis. J Am Coll Cardiol.

[7] Rapezzi C, Quarta CC, Guidalotti PL (2011). Usefulness and limitations of 99mTc-3,3-diphosphono-1,2-propanodicarboxylic acid scintigraphy in the aetiological diagnosis of amyloidotic cardiomyopathy. Eur J Nucl Med Mol Imaging.

[8] Bokhari S, Shahzad S, Castaño A, Maurer MS (2014). Nuclear imaging modalities for cardiac amyloidosis. J Nucl Cardiol.

[9] Glaudermans AW, van Rheenen RW, van den Berg MP (2014). Bone scintigraphy with (99m)technetium-hydroxymethylene diphosphonate allows early diagnosis of cardiac involvement in patients with transthyretin-derived systemic amyloidosis. Amyloid.

[10] Gillmore JD, Maurer MS, Falk RH (2016). Non-biopsy diagnosis of cardiac transthyretin amyloidosis. Circulation.

[11] Hutt DF, Quigley A-M, Page J (2014). Utility and limitations of 3,3-diphosphono-1,2-propanodicarboxylic acid scintigraphy in systemic amyloidosis. Eur Heart J.

[12] Coelho T, Maia LF, da Silva AM (2012). Tafamidis for transthyretin familial amyloid polyneuropathy. Neurology.

[13] Castaño A, Drachman BM, Judge D, Maurer MS (2015). Natural history and therapy of TTR-cardiac amyloidosis: emerging disease-modifying therapies from organ transplantation to stabilizer and silencer drugs. Heart Fail Rev.

[14] Benson MD, Pandy S, Witchell D, Jayazeri A, Siwkowski A, Monia B, Kluve-Bekerman B (2011). Antisense oligonucleotide therapy for TTR amyloidosis. Amyloid.

[15] Ramsay SC, Lindsay K, Fong W, Patford S, Younger J, Atherton J (2018). Tc-HDP quantitative SPECT/CT in transthyretin cardiac amyloid and the development of a reference interval for myocardial uptake in the non-affected population. Eur J Hybrid Imaging.

[16] Ross JC, Hutt DF, Burniston M (2018). Quantitation of ^99m^Tc-DPD uptake in patients with transthyretin-related cardiac amyloidosis. Amyloid.

[17] Caobelli F, Braun M, Haaf P, Wild D, Zellweger MJ (2019). Quantitative (99m)Tc-DPD SPECT/CT in patients with suspected ATTR cardiac amyloidosis: feasibility and correlation with visual scores. J Nucl Cardiol.

[18] Scully PR, Morris E, Patel KP (2020). DPD quantification in cardiac amyloidosis: a novel imaging biomarker. J Am Coll Cardiol Cardiovasc Imaging.

[19] Manrique A, Dudoignon D, Brun S (2019). Quantification of myocardial 99mTc-labeled bisphosphonate uptake with cadmium zinc telluride camera in patients with transthyretin-related cardiac amyloidosis. Eur J Nucl Med Mol Imaging Res.

[20] Bellevre D, Bailliez A, Delelis F (2020). Quantitation of myocardial ^99m^Tc-HMDP uptake with new SPECT/CT cadmium zinc telluride (CZT) camera in patients with transthyretin-related cardiac amyloidosis: ready for clinical use?. J Nucl Cardiol.

[21] Dorbala S, Park MA, Cuddy S (2013). Absolute quantitation of cardiac ^99m^Tc-pyrophosphate using cadmium zinc telluride-based SPECT/CT. J Nucl Med.

[22] Dorbala S, Ando Y, Bokhari S (2019). ASNC/AHA/ASE/EANM/HFSA/ISA/SCMR/SNMMI expert consensus recommendations for multimodality imaging in cardiac amyloidosis: part 1 of 2—evidence base and standardized methods of imaging. J Nucl Cardiol.

[23] Castano A, DeLuca A, Weinberg R (2016). Serial scanning with technetium pyrophosphate (^99m^Tc-PYP) in advanced ATTR cardiac amyloidosis. J Nucl Cardiol.

